# Starve or share? Phosphate availability shapes plant–microbe interactions

**DOI:** 10.1371/journal.ppat.1013601

**Published:** 2025-10-21

**Authors:** Maja Schmidt, Kiran Raj, Judith Salas-Oropeza, Oswaldo Valdés-López, Martina K. Ried-Lasi

**Affiliations:** 1 Department of Molecular Signal Processing, Leibniz Institute of Plant Biochemistry, Symbiosis Signalling Group, Halle (Saale), Germany; 2 Laboratorio de Genómica Funcional de Leguminosas, Facultad de Estudios Superiores Iztacala, Universidad Nacional Autónoma de México, Tlalnepantla, México; University of Tübingen: Eberhard Karls Universitat Tubingen, GERMANY

## How do plants monitor phosphate availability and respond to phosphate starvation?

**Fig 1:**
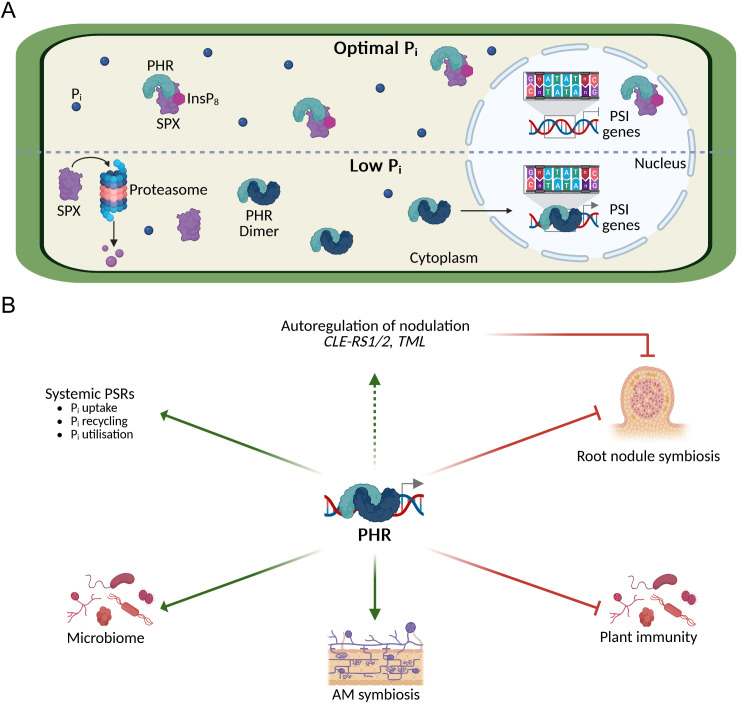
PHR regulates systemic phosphate starvation responses and the interaction with beneficial and detrimental microbes. **(A)** Upper panel: Under optimal P_i_ conditions, inositol pyrophosphates, particularly InsP_8_, bind to SPX proteins [15,17], promoting their interaction with PHR transcription factors [16,17]. This interaction inhibits PHR by preventing its nuclear localisation and/or DNA binding, thereby blocking access to PHR1 binding sequence (P1BS) promoter elements in the promoters of P_i_ starvation-induced (PSI) genes [18–22]. Lower panel: Under low P_i_ conditions, cellular InsP_8_ levels decline, triggering the proteasomal degradation of SPX proteins [20,66,67]. As a result, PHR is released, enabling the formation of PHR/PHL homo- and heterodimers [4,16,68], which bind to P1BS promoter elements and activate PSI gene expression. **(B)** Beyond its central role in activating systemic PSRs, PHR functions as a regulatory hub that integrates P_i_ status with biotic interactions positively (green arrows) or negatively (red lines) modulating gene expression, thus fine-tuning plant responses to environmental stimuli. Notably, PHR downregulates plant immune responses, thereby facilitating the restructuring of the root microbiome to favour beneficial microbial recruitment [61–63]. This function positions PHR as a critical genetic element enabling plants to attract microbes capable of enhancing P_i_ acquisition. In addition, PHR regulates genes involved in the establishment of AM, including elements of the CSSP, which is also required for root nodule symbiosis. Interestingly, in legumes such as *Phaseolus vulgaris* and soybean, PHR appears to negatively regulate root nodule symbiosis via activation of the AON pathway [57]. Candidate AON genes under PHR control include *CLAVATA3/ESR-related root signal 1* (*CLE-RS1*), *CLE-RS2*, and *Too Much Love* (*TML*) [57]. Created in BioRender. Valdés-López, O. (2026) https://BioRender.com/o1nfra8.

Phosphorus is an essential macronutrient that supports core biological processes such as photosynthesis, respiration, and the biosynthesis of nucleic acids and membranes [1]. Plants take up phosphorus from the soil as inorganic orthophosphate (P_i_) [2], yet P_i_ is poorly available in most soils due to its rapid fixation into insoluble complexes with iron and aluminium in acidic soils, and calcium in alkaline soils [3]. Consequently, P_i_ availability is a major limitation for plant growth and crop productivity [3]. To cope with P_i_ deficiency, plants have evolved a highly coordinated network of local and systemic phosphate starvation responses (PSRs) that are rapidly reversed upon P_i_ resupply. These PSRs involve morphological, transcriptional, and metabolic adjustments. Local responses primarily reshape root system architecture (e.g., inhibition of primary root growth, root hair formation and lateral root formation), systemic responses aim to maintain P_i_ homeostasis through improved P_i_ uptake, recycling, and utilisation [3]. In *Arabidopsis*, systemic PSRs are orchestrated by the MYB-type coiled-coil transcription factor Phosphate Response 1 (PHR1; [4]) and its homologues PHR1-likes (PHLs; [5–7], and orthologs have been described in several plant species [8–12] (Fig 1). These transcription factors activate P_i_ starvation-induced (PSI) genes by binding to the PHR1 Binding Sequence (P1BS) in their promoters [4] (Fig 1A). Among the targets are genes encoding high-affinity P_i_ transporters and enzymes involved in membrane phospholipids remodelling [3,5,13,14]. PHR activity is tightly regulated by SYG1/Pho81/XPR1 (SPX) domain-containing proteins. SPX domains act as high-affinity receptors for inositol pyrophosphates (PP-InsPs), which serve as proxies for cellular P_i_ status and mediate the interaction between SPX and PHR [15–17] thereby inhibiting PHR by sequestering it away from the nucleus or DNA [18–22] (Fig 1A). Interestingly, P_i_ signalling is not isolated but tightly interconnected with nitrogen status. In rice, under high nitrate conditions, the nitrate sensor Nitrate Transporter 1.1B (NRT1.1B; [23]) interacts with SPX4, promoting SPX4 degradation via the E3 ligase NRT1.1B interacting protein 1 (NBIP1; [24]). As a result, PHR2 and NIN-like protein 3 (NLP3; [25]) are released from SPX-mediated inhibition, translocate into the nucleus and activate PSI and nitrate-response genes, respectively [24]. In *Arabidopsis*, the expression of several PSI genes is reduced in an *nrt1.1* mutant and is influenced not only by P_i_ but also by nitrate availability [26]. Furthermore, PHR1 and NLPs regulate the expression of *Nitrate-Inducible GARP-type Transcriptional Repressor 1* (*NIGT1*) genes, which encode repressors of specific nitrate-response genes [27] as well as *SPX* genes [28]. These examples illustrate the tight interconnection between P_i_ and nitrate signalling, which has been comprehensively reviewed elsewhere [29,30]. MicroRNAs, particularly miR399 and miR827, add additional layers of regulation by downregulating negative regulators of P_i_ uptake, contributing to a robust and multi-tiered response system [31–35]. Notably, the SPX–PHR regulatory module is evolutionarily conserved across land plants, including early diverging lineages such as *Marchantia polymorpha*, highlighting its fundamental role in signalling [36].

## How does phosphate availability control arbuscular mycorrhiza?

Besides local and systemic PSRs, plants have evolved symbiotic associations with microorganisms to improve P_i_ uptake. Around 80% of land plants, including major crops such as rice, maize, and wheat, form arbuscular mycorrhiza (AM) with fungi belonging to the phylum Glomeromycota [37]. These mutualistic relationships emerged over 450 million years ago, supporting plant terrestrialization and possibly influencing historical atmospheric changes by enhancing CO₂ uptake and O₂ production [38]. AM is a type of endomycorrhiza connecting the plant root system to an extensive extraradical fungal network. AM fungi grow into plant roots and penetrate root cortical cells to form highly branched, intracellular structures called arbuscules, each surrounded by a plant-derived periarbuscular membrane [37,39]. Arbuscules are transient structures, degrading within days under tight plant and fungal control, and represent the main site for nutrient exchange [37,40]. Plants transfer up to 20% of their photosynthesis-derived carbon mainly in the form of fatty acids and sugars to the fungal partner in return for P_i_ and other essential nutrients [37,41–43], underscoring the importance of precisely tuning this symbiotic exchange according to P_i_ availability. This is further corroborated by the observation that AM is negatively regulated under sufficient P_i_ conditions [44,45]. The presence of P1BS cis-regulatory elements in the promoter regions of several AM-related genes has been known since 2011 [46,47], and mutations within these motifs strongly impair AM-induced promoter activity [46]. More recent studies have uncovered molecular links between P_i_ homeostasis and AM symbiosis mediated by the SPX–PHR signalling module and PP-InsP messengers [11,12,48–51]. In rice, for example, PHR2 functions as a central regulator of genes expressed in arbuscule-containing cells and primes roots for AM colonisation by directly targeting numerous symbiosis-related genes, even in the absence of the fungus [12,48]. These targets include genes encoding proteins involved in strigolactone biosynthesis, components of the common symbiosis signalling pathway (CSSP, [52]), which is shared with nitrogen-fixing root nodule symbiosis and crucial for both forms of plant root endosymbioses and nutrient transporters localised at the periarbuscular membrane [12,48]. The role of PHR transcription factors in the regulation of AM-related gene expression has further been corroborated in *Lotus japonicus* and *Medicago truncatula* [12,49]. Constitutive overexpression of *PHR* typically enhances AM colonisation in rice, *L. japonicus*, and *M. truncatula*, although effects depend on species, promoter choice, and P_i_ status [12,47,48]. Notably, in *M. truncatula*, using the *Ubiquitin1* promoter instead of the *35S* promoter to drive *MtPHR2* expression reduces AM fungal colonisation and accelerates arbuscule degradation [48]. Likewise, rice higher-order *spx* mutants as well as tomato *spx1* mutants display increased AM fungal colonisation, whereas *SPX* overexpression reduces AM development [47,49]. Conversely, *M. truncatula spx* mutants exhibit reduced AM development, while *SPX* overexpression increases AM fungal colonisation but promotes early arbuscule degeneration [11,48]. Species-specific differences are evident: rice upregulates *PHR2* expression in arbuscule-containing cells, whereas this does not occur in *M. truncatula* [47,48]. Conversely, *SPX* expression is arbuscule-induced in *M. truncatula* and tomato, but not in rice [11,47,49]. Recently, the Vip1-type diphosphoinositol pentakisphosphate kinase VIH2 was identified in *Lotus* as a key regulator of PP-InsP synthesis and AM; mutations in *VIH2* alter (PP)-InsP levels, enhancing AM fungal colonisation and P_i_ uptake across various external P_i_ concentrations [50]. These studies collectively illustrate how P_i_ availability modulates AM establishment, arbuscule formation, and maintenance through a conserved yet highly context-dependent regulatory network.

## Does phosphate availability control root nodule symbiosis?

In addition to AM, legumes establish mutualistic associations with nitrogen-fixing rhizobia bacteria [51]. This interaction also relies on the CSSP, which activates the genetic programs necessary for rhizobial infection and nodule organogenesis—specialised root organs that host nitrogen fixation [52]. Through this symbiosis, legumes can thrive in nitrogen-poor soils while enriching them with biologically fixed nitrogen. However, P_i_ deficiency strongly impairs root nodule symbiosis, reducing both the number of nodules and their nitrogen-fixing capacity by more than 50% across several legume species [53–55]. These observations raise a critical question: To what extent and how on a mechanistic level does the host plant’s P_i_ status determine the successful establishment of symbiosis with rhizobia? Recent studies provide evidence that P_i_ availability influences symbiotic success. Low P_i_ reduces the expression of core symbiotic genes [56] and activates the Autoregulation of Nodulation (AON) pathway, which normally limits nodule formation under sufficient P_i_ to conserve resources. Intriguingly, AON is also triggered by P_i_ deficiency in the absence of rhizobia, suggesting a preparatory mechanism that limits symbiosis when P_i_ is scarce [57]. Notably, several AON pathway genes (e.g., *Too Much Love*, *TML*; *CLE-Related-Root Signal 1* and *2*, *CLE-RS1* and *CLE-RS2*) contain the P1BS promoter element, suggesting that PHR integrates P_i_ signalling with symbiotic regulation [57]. Supporting this, experimental evidence indicates that both PHR and the AON pathway jointly modulate root nodule symbiosis under P_i_-deficient conditions [56,57]. Together, these findings underscore that the host plant’s P_i_ status plays an important role in regulating root nodule symbiosis, with PHR emerging as a key integrator of nutrient signalling and plant root endosymbioses.

## Was PHR recruited during evolution to link phosphate sensing with biotic interactions?

Land plants began colonising terrestrial environments over 450 million years ago, evolving mechanisms to conquer an inhospitable environment [58]. Among these challenges, low P_i_ availability was a key selective pressure, driving the emergence of molecular strategies to cope with P_i_ deficiency [59,60]. Phylogenomic analyses suggest that the PHR–SPX regulatory module, central to P_i_ signalling, originated in early green algae and diversified throughout plant evolution [36]. Promoter analyses in *M. polymorpha* further show that most P_i_-responsive genes contain at least one P1BS promoter element [36], underscoring the evolutionary conservation and functional relevance of this module in terrestrial adaptation. Beyond its role in canonical PSRs, PHR appears to function at the intersection of nutrient signalling and plant–microbe interactions (Fig 1B) [12,47,61,62]. In *Arabidopsis*, a non-host plant for AM fungi and rhizobia, PHR modulates root immunity and shapes microbiome composition, thereby promoting the recruitment of beneficial microbes that aid in plant adaptation to P_i_ deficiency or in controlling fungal infection, as demonstrated in beneficial interactions with *Colletotrichum tofieldiae* (Fig 1B) [61–64]. Similarly, the PHR–SPX module is required for the establishment and regulation of AM [11,12,47–49], and evidence from legumes like *Phaseolus vulgaris* and soybean indicates that PHR also contributes to the control of root nodule symbiosis in response to P_i_ availability (Fig 1B) [56,57]. Taken together, these findings support the hypothesis that during land plant evolution, the PHR–SPX module was co-opted not only for P_i_ homeostasis but also to facilitate symbiotic associations with soil microbes (Fig 1B). It is further conceivable that PHR shapes the root microbiome through the regulation of root exudates, thereby recruiting microbial partners that enhance P_i_ acquisition and support plant survival under nutrient stress.

## How does phosphate availability fine-tune beneficial and detrimental plant–microbe interactions?

As discussed above, P_i_ is essential not only for core metabolic functions but also for establishing root endosymbioses. PHR serves as a central integrator of plant P_i_ status, modulating responses to both nutrient availability and microbes. Originally, PHR has been described as a master regulator of systemic PSRs to mitigate P_i_ deficiency. Simultaneously, accumulating evidence shows that it shapes the root microbiome by promoting beneficial microbes and restricting associations with P_i_-demanding partners like rhizobia. Besides its canonical function, PHR appears to at least partially coordinate these processes by activating the expression of a set of transcription factors participating in chromatin accessibility and, thereby, allowing the activation of low-P_i_-responsive genes in *Arabidopsis* [65]. A mechanistic understanding of how P_i_ availability and thus the PHR–SPX module regulates beneficial as well as detrimental plant microbe interactions is an important avenue for future research. Together, these findings suggest that the PHR–SPX module is part of an ancient regulatory toolkit co-opted to fine-tune biotic interactions in response to environmental nutrient cues.
